# Identification of Host Insulin Binding Sites on *Schistosoma japonicum* Insulin Receptors

**DOI:** 10.1371/journal.pone.0159704

**Published:** 2016-07-21

**Authors:** Rachel J. Stephenson, Istvan Toth, Jiening Liang, Amanjot Mangat, Donald P. McManus, Hong You

**Affiliations:** 1 School of Chemistry and Molecular Biosciences, The University of Queensland, St. Lucia, QLD, Australia; 2 School of Pharmacy, The University of Queensland, Woolloongabba, QLD, Australia; 3 Institute for Molecular Bioscience, The University of Queensland, Brisbane, Australia; 4 Molecular Parasitology Laboratory, Infectious Diseases Division, QIMR Berghofer Medical Research Institute, Brisbane, Queensland, Australia; Universidade Guarulhos, BRAZIL

## Abstract

*Schistosoma japonicum* insulin receptors (SjIRs) have been identified as encouraging vaccine candidates. Interrupting or blocking the binding between host insulin and the schistosome insulin receptors (IRs) may result in reduced glucose uptake leading to starvation and stunting of worms with a reduction in egg output. To further understand how schistosomes are able to exploit host insulin for development and growth, and whether these parasites and their mammalian hosts compete for the same insulin source, we identified insulin binding sites on the SjIRs. Based on sequence analysis and the predicted antigenic structure of the primary sequences of the SjIRs, we designed nine and eleven peptide analogues from SjIR-1 and SjIR-2, respectively. Using the Octet RED system, we identified analogues derived from SjIR-1 (10) and SjIR-2 (20, 21 and 22) with insulin-binding sequences specific for *S*. *japonicum*. Nevertheless, the human insulin receptor (HIR) may compete with the SjIRs in binding human insulin in other positions which are important for HIR binding to insulin. However, no binding occurred between insulin and parasite analogues derived from SjIR-1 (2, 7 and 8) and SjIR-2 (14, 16 and 18) at the same locations as HIR sequences which have been shown to have strong insulin binding affinities. Importantly, we found two analogues (1 and 3), derived from SjIR-1, and two analogues (13 and 15) derived from SjIR-2, were responsible for the major insulin binding affinity in *S*. *japonicum*. These peptide analogues were shown to have more than 10 times (in KD value) stronger binding capacity for human insulin compared with peptides derived from the HIR in the same sequence positions. Paradoxically, analogues 1, 3, 13 and 15 do not appear to contain major antigenic determinants which resulted in poor antibody responses to native *S*. *japonicum* protein. This argues against their future development as peptide-vaccine candidates.

## Introduction

Schistosomiasis remains one of the most prevalent and chronically serious tropical parasitic diseases with an estimated 240 million people infected in 78 countries, and close to 800 million are at risk [1]. It has been reported that at least 258 million people required preventive treatment with the drug paraziquantel (PZQ) in 2014 [1]. The causative schistosome bloodflukes represent a significant health problem affecting the health and intellectual capacity of infected individuals, and as a result are linked directly or indirectly to hundreds of thousands of deaths annually [2]. PZQ chemotherapy, available for more than three decades, has reduced morbidity rates but the consequences of continuous re-infection in endemic areas remain unchanged [3]. Additionally, mass use of this compound has led to concern for the emergence of PZQ-resistant parasites [4]. Vaccines represent the most attractive long-term alternative to invert this scenario [4–6]. Despite the discovery and publication of numerous potentially promising vaccine antigens from *Schistosoma mansoni* and *S*. *haematobium*, only three molecules, *S*. *mansoni* fatty acid binding protein (Sm14), *S*. *mansoni* tetraspanin-2 (Sm-TSP-2) and *S*. *haematobium* glutathione S-transferase (Sh28GST) have entered human clinical trials [7]. Progress in the development of an effective vaccine against *S*. *japonicum* has been even more disappointing. Nevertheless, a vaccine capable of reducing worm burdens and/or faecal egg output has historically been considered the “ultimate goal” and, in conjunction with PZQ treatment, has the potential to lead to a significant reduction in *S*. *japonicum* transmission, almost to the point of elimination [8]. Based on the fact that schistosome eggs are responsible for both pathology and transmission, and that female worms of *S*. *japonicum* produce a substantially higher daily egg output compared with other schistosome species, development of a transmission blocking vaccine against *S*. *japonicum* targeting parasite fecundity and egg viability is entirely relevant [9].

*S*. *japonicum* insulin receptors (IRs) have been identified as encouraging transmission blocking vaccine candidates [9], playing an important role in parasite growth and fecundity (egg production) through their involvement in glucose metabolism. It has been well recognized that schistosomes consume their dry weight of glucose (obtained from their mammalian hosts) every 5 hours [10]. Previous studies have demonstrated that two types of IRs present in *S*. *japonicum* (SjIR-1 and 2) [11] and *S*. *mansoni* (SmIR-1 and 2) [12] can bind to human insulin, and this interaction can activate the downstream signalling transduction of tyrosine kinase, thereby regulating glucose uptake from the host by these parasites [9,13]. Previous studies of ours and others on schistosome IRs have shown that IR-1, which is located on the surface of adult worms, may be more involved in utilizing host insulin than IR-2 [11,12]. In addition to this, the diffuse expression of schistosome IR-2 observed in the parenchyma of adult males and in the vitelline cells of females argues that it has a possible function in the control of growth, similar to that described for the HIR [11]. We have shown that disruption of the insulin pathway in schistosomes by knocking down the SjIRs *in vitro* resulted in reduced glucose uptake leading to starvation and stunting of worms with a reduction in egg output [9,13]. This outcome was supported by recent vaccine/challenge trials we undertook whereby immunization with the ligand domain of the SjIRs fusion proteins induced a significant retardation in worm growth (12-42% reduction in worm lengths), depressed fecundity (50-67% faecal egg reduction) and a reduction in the numbers of mature intestinal eggs (75%) in a murine vaccine/cercariae challenge model [9,13].

However, to clarify how schistosomes exploit host insulin and how the mammalian host and these parasites compete for the same insulin source, further studies are required to identify parasite-specific insulin binding epitopes which may be evaluated as potential peptide-vaccine candidates. Our previous data have shown that schistosome IRs belong to a family of IRs containing a conserved catalytic domain, sharing a conserved α2β2 heterotetramer structure with the IRs of *Echinococcus multilocularis*, *Drosophila* and *Homo sapiens* [11]. Schistosome IRs exhibit most of the features of other IR family members in both the extracellular ligand domain (LD) which may contain insulin binding sites within two characteristic loops (L1 and L2) flanked by a central cysteine-rich (CR) region, and intracellular tyrosine kinase (TK) domain which is very conserved and has been shown to be activated by the binding between insulin and IR for downstream signalling transduction.[14]

Recently, we have shown that the LD of the SjIR-1 and 2 fusion proteins can strongly bind to human insulin [13]. While the interaction of human insulin with its receptor is complex, the essential amino acid residues which allow the receptor to adopt the correct conformation and enable insulin binding have been the subject of extensive investigation [15]. The human insulin receptor (HIR) has receptor binding sites located in the L1 sub-domain and the first and second type fibronectin III repeats (FnIII-1 and FnIII-2), respectively [16]. The closure of the L1 unit of one monomer of HIR towards the FnIII-1 domain of the other monomer would also drag the FnIII-2, 3 unit with it, thereby changing the relative position of the intracellular kinase domains in the IR dimer and potentially inducing downstream signalling transduction [17]. Alanine scanning of this L1 sub-domain indicated a discontinuous binding site where Arg14, Asn15, and Phe64 were shown to be essential for binding, with minor contributions from other peripheral amino acids. The second L1 domain binding sequence is located in the bulge between the first and second beta-sheets of the beta-helix and consists of Leu87-Try91. Further, in the FnIII domain, residues Leu736-Phe741 are important for insulin binding activity.[15] Using this information, together with phylogenetic, sequence and three-dimensional structural analysis determined by comparative homology modelling [11], we aimed to identify insulin binding sequences on the L1, L2, FnIII-1 and 2 sub-domains of SjIR-1 and 2. This was achieved through the synthesis of peptides derived from SjIR primary sequences with the potential to bind insulin, based on previous insulin binding sites identified in the HIR.

## Materials and Methods

### Peptide design

Several criteria were used to identify peptide segments as potential insulin binding candidates in *S*. *japonicum*. These included: (i) identification of potential regions of antigenicity on the SjIR proteins using a proprietary OptimumAntigen™ design tool (GenScript Biotech Corporation, Piscataway, New Jersey, USA); (ii) selection of segments in the SjIR proteins that had significant sequence conservation with *S*. *mansoni* and *E*. *multilocularis* to better identify parasite-specific insulin binding sites, and (iii) ensuring minimal conservation/homology with the HIR sequence[5]. Peptide sequences with low homology to the HIR but permitting high conservation between the parasite sequences, were selected. Furthermore, in choosing the exact length and location of each peptide segment, we attempted to maintain the peptide sequences to the minimum possible length to ensure that the peptides were not too short, thereby missing critical residues, or too long, resulting in synthesis failure.[18–21]

**Fig 1:**
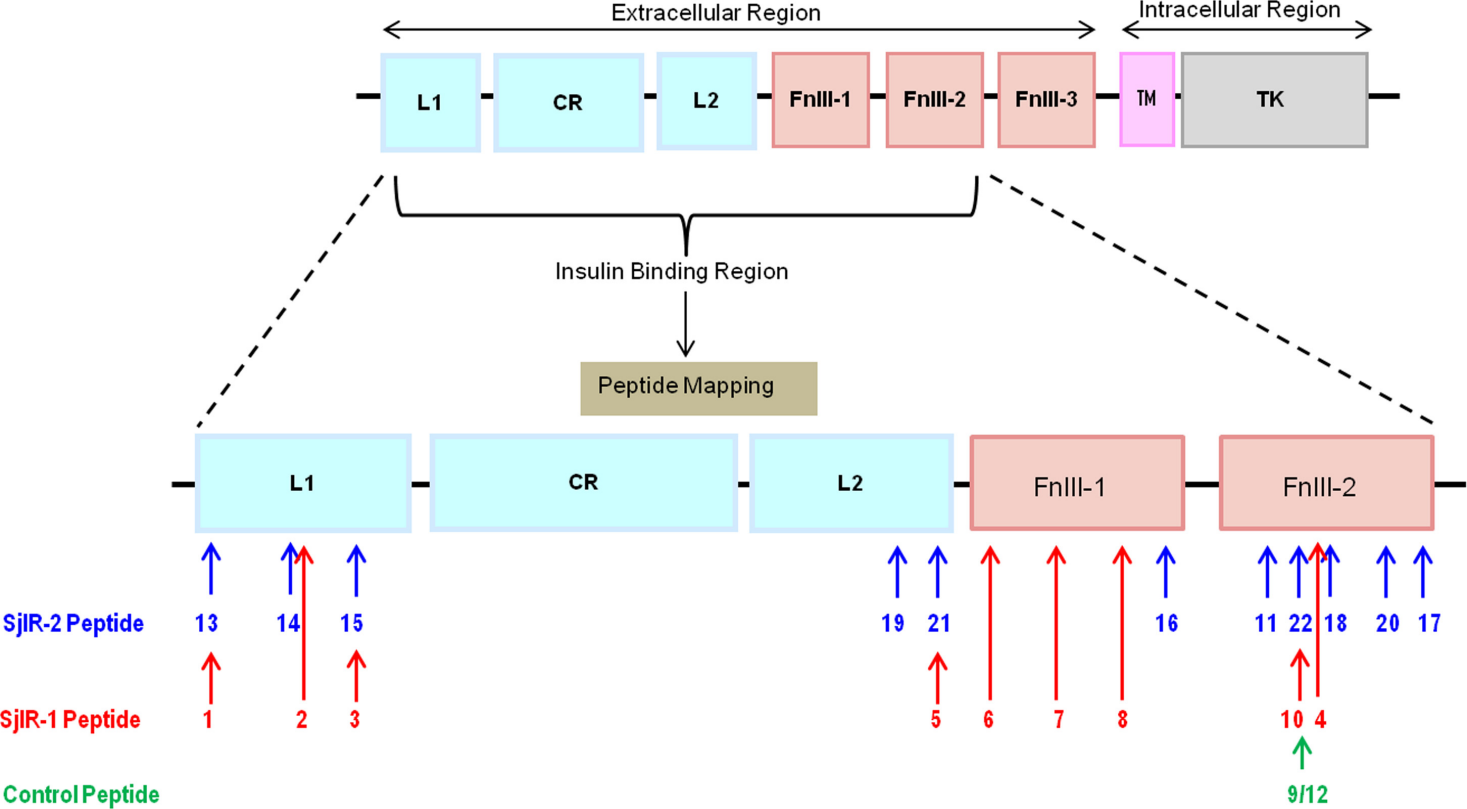
Schematic model of the SjIRs with approximate locations of mapped peptide analogues 1-22. Peptides 1-10 (in red) are located on SjIR-1; peptides 11, 13-22 (in blue) are located on SjIR-2. Peptide 9 is located on the HIR (its approximate location relative to the sequence in the SjIRs is indicated). Peptide 12 is the reverse analogue of peptide 9. L1, L1 subdomain; L2, L2 subdomain; CR, cysteine rich domain; FnIII-1-3, fibronectin domains 1, 2 and 3; TM, transmembrane domain; TK, tyrosine kinase domain.

On the basis of this sequence analysis, together with the predicted protein structure, nine peptide analogues from SjIR-1 and eleven peptide analogues from SjIR-2 were identified (Table 1, Fig 1). Analogues 1-3 and 13-15 are located in the L1 domain, analogues 5, 19 and 21 are located in the L2 domain, analogous 6-8 and 16 are located in the FnIII-1 domain, and analogues 4, 9-12, 17-18 and 20, 22 are located in the FnIII-2 domain (Table 1, Fig 1). The positive control (analogue 9, Table 1), from the alpha subunit of the HIR (α655-670), has been found to exhibit specific binding activity towards radioiodinated insulin.[22] Analogue 12 is identical in amino acid composition to analogue 9 but its sequence has been reversed, and has been designed as a control in the binding assays to evaluate the non-specific effect of a peptide of unrelated amino acid sequence but identical amino acid content. All peptide sequences are presented in Table 1. Alignment of multiple sequences of IRs from *S*. *japonicum*, *S*. *mansoni*, *E*. *multilocularis*, *Drosophila*, and *Homo sapiens* and the positions of all the analogues designed for this study are provided in the Supporting Information as S1 Fig.

**Table 1:** Details of peptide analogues screened for insulin binding activity including sequence information and their location within the SjIR proteins.

Analogue	IR Domain	Sequence	Identity to SmIRs or HIR	Sequence Information	Reference
1	SjIR-1 L1	NLRESNNLSSLTNCSTIHGTLVIRN	68% (SmIR-1) 36% (HIR)	• Same location as the HIR epitope containing amino acids important for insulin binding (Asp12, Arg14, Asn15; Gln34, Leu36, Leu37) with Arg12 and Leu36 being conserved for all sequences analyzed (see S1 Fig) • Same sequence location as analogue 13 from SjIR-2	[15]
2		GSLIIENGNCSGDLSTLLPNLTVIRNQVI	76% (SmIR-1) 40% (HIR)	• Same location as the HIR epitope containing amino acid Phe64 which has been shown to be important for insulin binding to the HIR	[15]
3		SDYSLIIRHTKLKGIGLWKLKTLNSYPIALIDNPLMC	73% (SmIR-1) 22% (HIR)	• Same location as the HIR epitope containing amino acids important for insulin binding (Phe64; Val94, Glu97; Glu120, Lys121,and Phe89-Try91). No amino acids from these epitopes have been shown to be conserved for all sequences analyzed, however, Try91 and Val94 was moderately conserved	[15]
5	SjIR-1 FnIII-1	MFTLNRNLCPNDV	63% (SmIR-1) 23% (HIR)	• Same sequence location as analogue 21 from SjIR-2	[23,24]
6		NLSNLEFDLIEKSNG	90% (SmIR-1) 27% (HIR)	• Highly antigenic region with low homology to the HIR	
7		IGIDESESIIETIC	71% (SmIR-1) 14% (HIR)	• Same sequence location as the HIR α655-670 which showed strong binding with insulin	
8		HEPKCHKVIEPITN	71% (SmIR-1) 14% (HIR)	• Same sequence location as the HIR α655-670 which showed strong binding with insulin	
9	HIR FnIII-2	SRTWSPPFESEDSQKH	25% (SjIR-1) 12.5% (SjIR-2)	• HIR (positive control) designed to test the binding between the HIR and insulin	[25]
4	SjIR-1 FnIII-2	TLLRLNTIQGSIFN	57% (SmIR-1) 17% (HIR)		
10		RLMSLPTMSNGNTVNN	25% (HIR) 31% (SmIR-1)	Same position of Analogue 9	
11	SjIR-2 FnIII-2	QSGWDKNSLFKHSRQI	47% (SmIR-2) 13% (HIR)	Same position of Analogue 9	
12	-	HKQSDESEFPPSWTRS		• Reversed sequence of analogue 9 (negative control)	
13	SjIR-2 L1 domain	ADVRHSSSLTKLSRCTVIEGDLFIVFTR	86% (SmIR-2) 39% (HIR)	• Same location as the HIR epitope containing amino acids important for insulin binding (Asp12, Arg14, Asn15; Gln34, Leu36, Leu37). Arg12 and Leu36 are conserved for all sequences analyzed • Same sequence location as analogue 1 from SjIR-1	[15]
14	SjIR-2 L1	IPRDASLPFLKEVTGSLLVYDTEGPEDL	86% (SjIR-1) 39% (HIR)	• Same location as the HIR epitope containing amino acid Phe64, which has been shown to be important for insulin binding to the HIR	[15]
15	SjIR-2 L1	LVFGYSVVIKSTSFKSIGLPSLRVIQQGGVRIDSNPQLC	90% (SmIR-2) 41% (HIR)	• Same location as the HIR epitope containing amino acids important for insulin binding (Phe64; Val94, Glu97; Glu120, Lys121,and Leu87-Try91). No amino acids from this epitope was shown to be conserved for all sequences analyzed, however, Phe88, Try91 and Val94 was moderately conserved (see SI) • Same position as analogue 3 in SjIR-1	[15]
16	SjIR-2 FnIII-1	AYLIWIRILEDNPSEY	88% (SmIR-2) 25% (HIR)	• Same sequence location as the HIR α655-670, which showed strong binding with insulin	
17	SjIR-2 FnIII-2	PRNHDQSYTDSNHS	48% (SmIR-2) N/A (HIR)	• Highly antigenic region with very low homology to HIR	
18		ILPVIVDEVVSLKSDTVG	68% (SmIR-2) 22% (HIR)	• Same location as the HIR epitope containing amino acids important for insulin binding (Leu736-Asn138). No amino acids from these epitopes are conserved for all sequences analyzed, however, Leu736 was moderately conserved • Highly antigenic region with low homology to the HIR	[15]
19	SjIR-2 L2	AKDANNIEDDPVN	46% (SmIR-2) 22% (HIR)	• Highly antigenic region with low homology to HIR	
20	SjIR-2 FnIII-2	ESETKCHRPPPWSN	50% (SmIR-2) N/A (HIR)	• Highly antigenic region with very low homology to HIR	
21	SjIR-2 L2	IRITQNRQLCPEKI	100% (SmIR-2) 29% (HIR)	• Highly antigenic region with low homology to HIRSame sequence location as analogue 5 from SjIR-1	
22	SjIR-2 FnIII-2	DFCTHRPNWIQSGWDKNS	66% (SmIR-2) 17% (HIR)	• Highly antigenic region with low homology to HIR	
28	HIR	MDIRNNLTRLHELENCSVIEGHLQILLMF	36% (SjIR-1) 32% (SjIR-2)	• Same sequence location as analogues 1 and 3	
29	HIR	LFFNYALVIFEMVHLKELGLYNLMNITRGSVRIEKNNELC	21% (SjIR-1) 36% (SjIR-2)	• Same sequence location as analogues 13 and 15	

N/A means no sequence homology or less than 10% identity; HIR, human insulin receptor; Sm, *S*. *mansoni*.

### General reagents and methods

Dimethylformamide (DMF), trifluoroacetic acid (TFA), and piperidine of peptide synthesis grade was purchased from Merck Biosciences (Kilsyth, VIC, Australia). HPLC-grade acetonitrile (MeCN) was purchased from RCI Labscan Ltd. (Bangkok, Thailand). Fmoc-protected amino acids and Rink amide MBHA resin (100–200 mesh, 0.4–0.8 mmol/g loading) was obtained from Novabiochem (Melbourne, VIC, Australia) or Mimotopes (Clayton, VIC, Australia). Microwave assisted Fmoc SPPS was carried out by using a SPS mode CEM Discovery reactor (CME Corporation, Matthews, NC, USA). Preparative HPLC was carried out on a Shimadzu system equipped with a CBM-20A controller, LC-20AT pump, SIL-10A autosampler, SPD-20A UV/Vis detector set to a wavelength of 230 nm and a FRC-10A fraction collector. The analytical HPLC was a Shimadzu instrument with an LC-20AB pump, a SIL-20AHT autosampler and an SPD-M10A detector set to a wavelength of 214 nm. Electrospray ionization mass spectrometry (ESI-MS) was performed on a PE Sciex API3000 triple quadrupole mass spectrometer, operating with a constant flow of a 1:1 mixture of solvent A (0.1% formic acid in water) and B (0.1% formic acid in acetonitrile/water 9:1) at a rate of 0.05 mL/min.

Protein binding assays were carried out on the Octet RED system (ForteBio, Menlo Park, CA, USA). Streptavidin Biosensors were obtained from ForteBio. Human insulin was biotinylated using a NHS-PEO4-biotin kit (Thermo Scientific, Rockford, IL, USA).

### Peptide synthesis

#### Microwave solid-phase peptide synthesis

Peptides (1-22) were assembled on Rink amide MBHA resin (0.2 mmol scale) using the *in situ* neutralization protocol for microwave-assisted Fmoc solid phase peptide synthesis (SPPS) [26]. N-Fmoc-protected amino acids (4.2 eq.), excluding Arg and His (4.2 eq.), were activated with HATU (4 eq.) and DIPEA (5 eq.) and coupled twice to the resin (2 x 5 min, 70°C, 20 Watt). Fmoc amino acids Arg and His (4.2 eq.) were activated with HATU (4 eq.) and DIPEA (5 eq.) and coupled twice to the resin (R.T x 5 min then 50°C x 5 min, 20 Watt). The Fmoc protecting group was removed by treatment with 20% (v/v) piperidine in DMF (2 x 2 min, 50°C, 20 Watt). Once the peptide sequence was complete, the resin was washed with DMF, MeOH and DCM, and dried *in vacuo*. The peptide was cleaved by stirring the resin in a mixture of TFA (95%), water (2.5%) and triisopropyl silane (2.5%) for 3 hr. Addition of cold diethyl ether precipitated the peptide, the solvent was discarded, and the peptide was dissolved in a mixture of acetonitrile and water (1:1) containing 0.1% TFA and lyophilized.

#### Purification

Crude peptides were purified by preparative RP-HPLC using a Vydac C18 column (22 mm × 250 mm) with a gradient of 100% A (water, 0.1% TFA) to 100% B (90% acetonitrile in water, 0.1% TFA) over 60 min at a flow rate of 20 ml/min. The collected fractions were analyzed by analytical HPLC using a Vydac C18 (4.6 mm × 250 mm, 5 μm) and a Vydac C8 column (4.6 mm × 250 mm, 5 μm) with a gradient of 100% A to 100% B over 30 min at a flow rate of 1 ml/min. Fractions containing pure peptide were combined and lyophilized.

#### Characterization

Characterization of peptides 1-22 by RP-HPLC and ESI-MS and their purification yields are shown in the Supporting Information (S1 Table).

### Insulin binding assays

Binding assays between human insulin and the synthesized peptides were performed using the Octet Red system (FortéBio, Menlo Park, CA, USA) in 96-well microplates at 25°C as described [13]. Briefly, human insulin was biotinylated using a NHS-PEO4-biotin kit (Thermo scientific, Rockford, IL, USA). Assays were carried out by placing the Streptavidin Biosensors (ForteBio) in the wells and measuring changes in layer thickness (nm) over time (sec). Firstly, a duplicate set of sensors were rinsed in kinetic buffer (1 mM phosphate, 15 mM NaCl, 0.1 mg/ml BSA, 0.002% Tween-20) for 300 sec which served as the baseline. Secondly, sensors were immobilized for 600 sec with 200 μl culture containing biotinylated human insulin (25μg/ml). Thirdly, sensors were washed in kinetic buffer for another 600 sec. Finally, sensors were exposed to a series of diluted samples run in 200 μl volumes in the same assay. Analogue 9, derived from the HIR and has known binding affinity to the HIR, was used as a positive control [23,24]. Analogue 12 (reversed sequence of analogue 9) was used as a negative control. The association of each analogue with insulin was monitored for 1000 sec followed by dissociation in kinetic buffer for 1000 sec. Data analysis from the FortéBio Octet RED instrument included a double reference subtraction. Sample subtraction was performed using BSA as a reference control, and sensor subtractio was performed on all samples automatically using Octet User Software 7 [27].

### Secondary structure analysis

Secondary structure analysis, using a Jasco J-710 Circular Dichroism spectrometer (Jasco J- 710), was undertaken to determine if SjIR peptide analogues 1, 3, 13 and 15, which exhibited strong binding to human insulin in the earlier Octet RED experiments, contained any predicted secondary structures. Peptides were dissolved in water (1 mg/mL) and the solution was transferred into a low volume 1.0 mm quartz cuvette. Peptide conformation studies were carried out at a wavelength range of 260 nm to 190 nm. All analysis was performed in triplicate and averaged. Spectra were normalized and reported as mean residual ellipticity (θ) versus wavelength. Additionally, previously generated homology models of the SjIR-1 and 2 receptor proteins [11] were used to predict the secondary structure of peptide analogues 1, 3, 13, and 15. Structural comparisons and figure generation were achieved using PyMOL Molecular Graphics System Version 1.5.0.4 (Schrödinger, LLC).

### Antibody generation and Western blot analysis

Antibodies were raised commercially against peptide analogues 1, 3, 13, and 15 in mice by GensCript Biotech Corporation (Piscataway, New Jersey, USA). Briefly, mice received a primary immunization with 50 μg peptide conjugated with ovalbumin. Three boosts at three weekly intervals were commenced two weeks after the primary immunization. The injections were delivered subcutaneously at multiple sites along the neck and spine. Blood was collected two weeks after the final boost. The titre of the antibody was determined using an enzyme-linked immunosorbent assay (ELISA). Briefly, Maxisorb immunoplates (Nalge Nune International, USA) were coated overnight at 4°C in coating buffer (100 μl /well) with peptide (100 μl of 4 μg/ml) or adult worm antigen preparation (SWAP) from *S*. *japonicum* (see below). To measure titres of antibodies generated in mice against individual peptides derived from analogues 1, 13, 3, and 15, conjugated with ovalbumin, each of the analogues (without conjugated ovalbumin) was coated on plates. After three washes with 0.05% (v/v) Tween in PBS (PBST), wells were blocked with 200 μl of 5% (v/v) skim milk in PBS (SMP) and incubated for 1 h at 37°C. The mouse anti-peptide serum was serially diluted (from 1:50 to 1:25,600) in SMP and 100 μl in duplicate of each dilution were added to individual wells. After incubation at 37°C for 1 h, the wells were washed with PBST (3X) and 100 μl (1:2,000 dilution) of horseradish peroxidise (HRP)-conjugated goat anti-mouse IgG (Invitrogen) was added. After incubation at 37°C for 1 h, the wells were washed with PBST (5X), 100 μl of substrate solution [2,2-azino-di-(ethyl-benzithiozolin sulfonate)] (Sigma, Castle Hill, Australia) was added, the wells were incubated at room temperature for 20 mins and read on a plate reader using Microplate manager software (Bio-Rad, Mississauga, Canada). Data are presented as antibody endpoint titres, defined as the highest dilution of test serum that yielded an average O.D. two standard deviations (SDs) greater than that obtained in the absence of primary antibody.

The mouse anti-peptide serum was used in Western blotting to probe the native protein in a separated crude *S*. *japonicum* antigen extract. The crude antigen was prepared from adult worms of *S*. *japonicum* freshly perfused from the intestinal veins of mice percutaneously infected with 60 cercariae six weeks previously. After three washes in perfusion buffer (8.5 g NaCl and 15 g NaCitrate in 1 l of water), to minimise contamination of the schistosome protein extract with host components, an adult worm antigen preparation (SWAP) was made as described [11]. The SWAP samples were separated on a 15% (w/v) SDS-PAGE gel and transferred to an Immun-Blot® low fluorescence-PVDF membrane. Overnight blocking was performed with Odyssey buffer containing 3% (v/v) goat serum at 4°C. Then, the membrane was subjected to incubation with the mouse anti-peptide anti-serum (1:100 dilution in Odyssey buffer and 0.1% Tween-20) for 1 h followed by incubation with IRDye-labeled 680LT goat anti-rabbit IgG antibody (Li-COR Biosciences) (1:15,000 diluted in Odyssey buffer with 0.1% Tween-20 and 0.01% SDS) for 1 h on a shaker in a dark chamber. After a final wash with distilled water, the membrane was allowed to dry in the dark and visualized using the Odyssey® CLx Infrared Imaging System [28].

## Results and Discussion

### Peptide Design and Synthesis

To date, IRs have been characterized from only three flatworm species: *S*. *japonicum* [11], *S*. *mansoni* [12] and *E*. *multilcularis* [29]. As a recognised model organism, *Drosophila melanogaster* IR [30] has been much studied. We compared IR sequence and binding site information from *S*. *japonicum*, *S*. *mansoni*, *D*. *melanogaster* and *Homo sapiens* [31] and used this information for the the design of our peptide analogues (S1 Fig). In order to identify parasite-specific insulin binding sites on SjIR-1 and 2, we synthesized a library of nine and 11 peptides, respectively, with an emphasis being placed on the extracellular regions of the IR proteins (L1 through to the FnIII-2 domain, Fig 1) [16]. Peptides were chosen with relatively low sequence similarity level to the HIR but high conservation amongst other helminth sequences (Table 1, S1 Fig).

Peptide analogue 13 was used as a representative example to illustrate peptide purity and quality. As shown in S2 Fig, a single distinct peak was found in the RP-HPLC chromatogram indicating that the peptide was highly pure. Furthermore, a molecular mass demarcated by a [M+2H]^+2^ 1576.9 peak was identified in the mass spectrum (calculated: 1576.33). RP-HPLC and mass spectrum results for the 24 peptides (analogues 1-22 and 28-29) were acquired with an average purity of 98%, as determined by RP-HPLC and MS.

### *In Vitro* Evaluation

**Fig 2:**
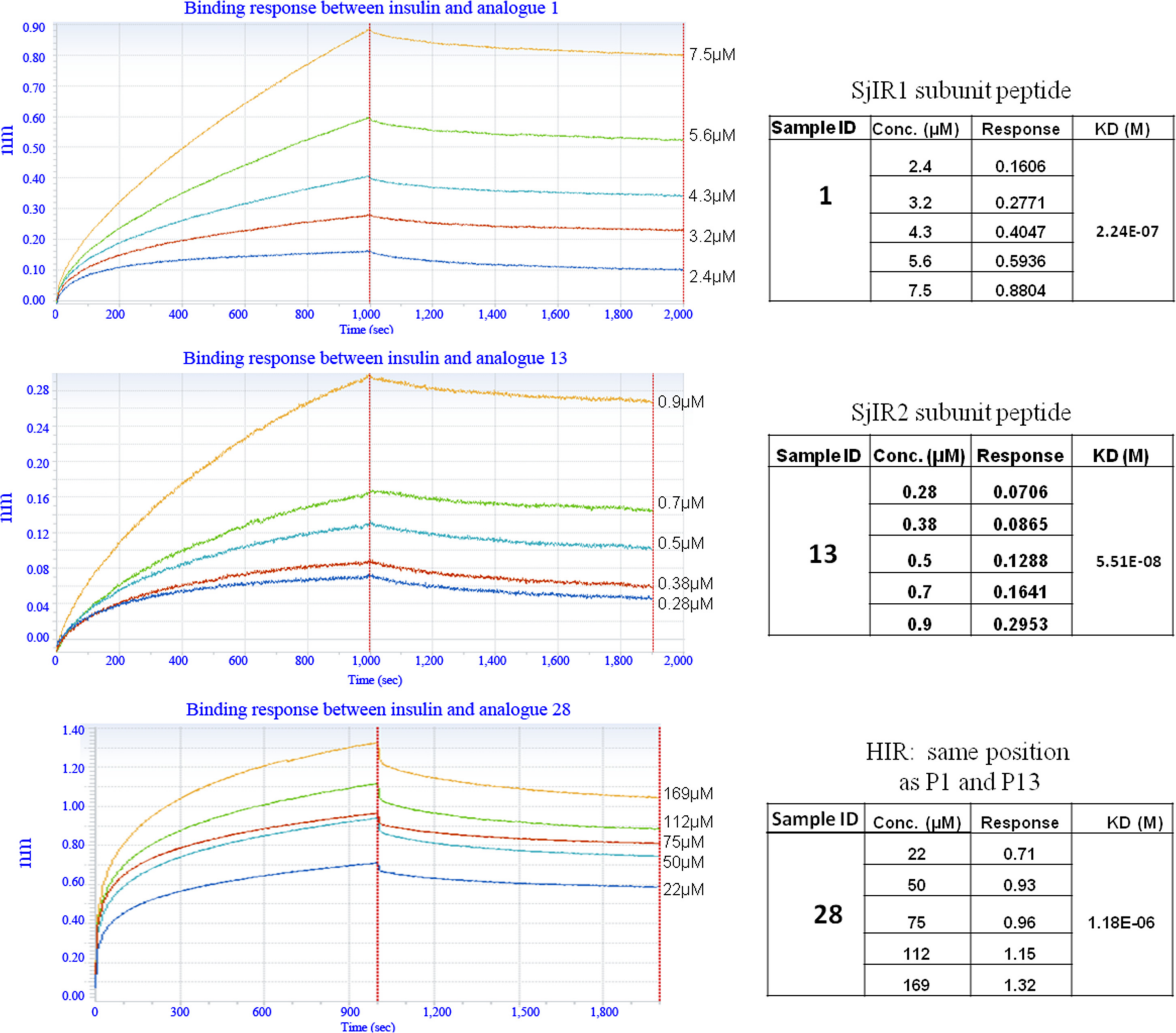
The binding affinity between human insulin and analogues 1, 13 and 28 derived from SjIR-1, SjIR-2 and HIR, respectively, determined using the Octet RED system. Binding is shown between human insulin and analogues 1 (A), 13 (B) and 28 (C) at different peptide concentrations (μM). The real time binding response (nm) was measured in seconds (sec). The parameters of the binding response (nm) and the KD value (M) of the binding between insulin and the analogues at different concentrations are shown. The coefficient of determination (R^2) of these interactions was close to 1.0, indicating a good curve fit. The left panels show that an increased binding response was observed when the concentration of peptide analogues was increased from low to high, as shown on the right in the tables.

**Fig 3:**
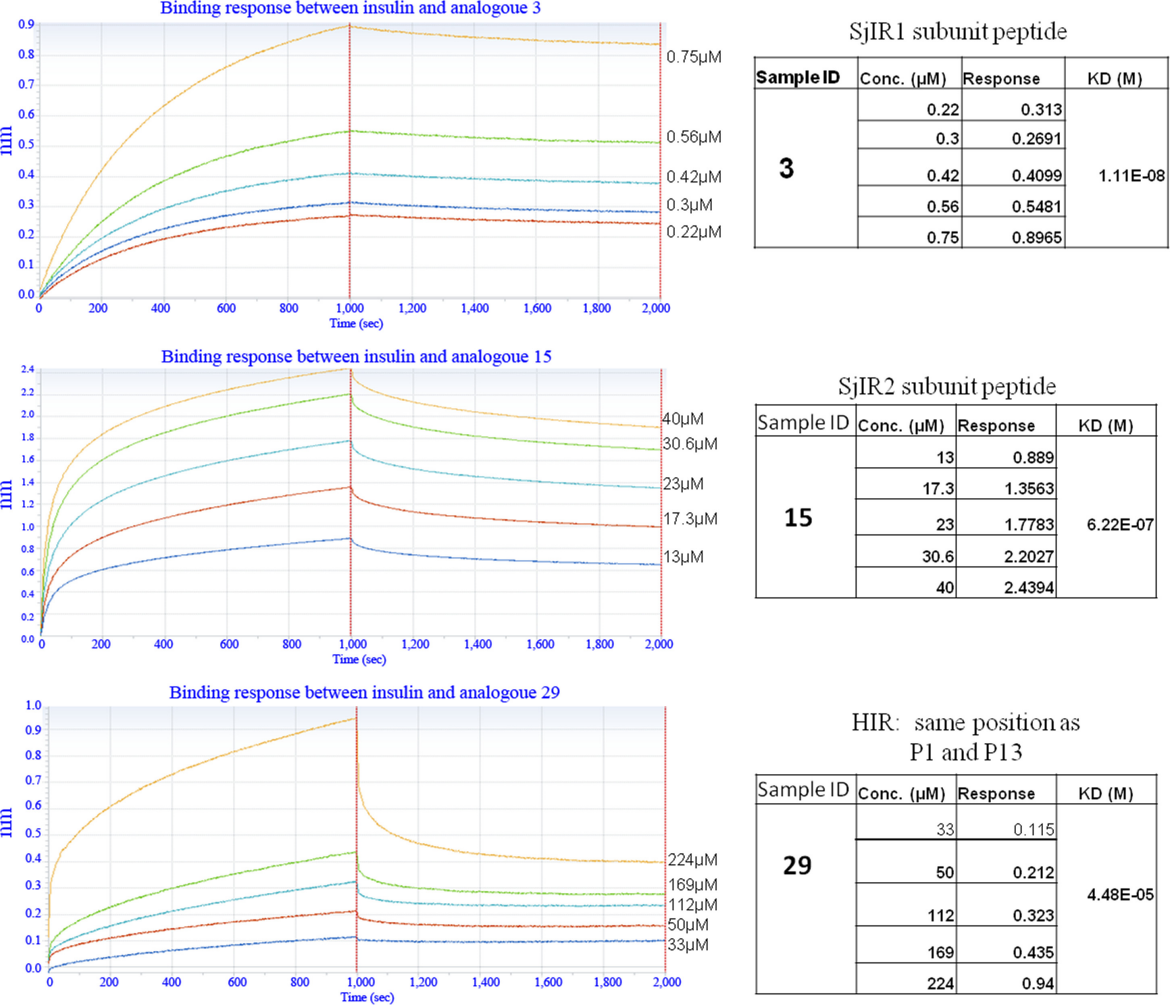
The binding affinity between human insulin and analogues 3, 15 and 29 derived from SjIR-1, SjIR-2 and HIR, respectively, measured using the Octet RED system. Binding between human insulin and analogues 3 (A), 15 (B) and 29 (C) is shown at different concentrations (μM). The real time binding response (nm) was measured in seconds (sec). The parameters of the binding response (nm) and the KD value (M) of the binding between insulin and the analogues at different concentrations are shown in the tables on the right. The coefficient of determination (R^2) for all these interactions was close to 1.0, indicating a good curve fit.

Octet RED technology was used to measure the binding affinity between human insulin and all the synthesized peptides presented in Table 1. Real time analysis using this system showed that there was specific interaction *in vitro* between human insulin and some of the peptide analogues, including 1, 13, 3, 15, 21, 20, 10, and 22 (Table 2). Increasing the concentration of the peptides in this experiment led to an increased binding response (Figs 2 and 3) with the dissociation phase slowly decreasing. This is indicative of a specific interaction during the association phase. As a result, we identified three insulin binding sites on SjIR-1 and five insulin binding sites on SjIR-2. These included major insulin binding sites I (located at the position of analogue 1 from SjIR-1 and analogue 13 from SjIR-2) and II (located at the position of analogue 3 from SjIR-1 and analogue 15 from SjIR-2). Analogue 1 from SjIR-1 and analogue 13 from SjIR-2 (Table 1) strongly bound insulin (Table 2), exhibiting KD values of 2.2E-07 and 5.51E-08, respectively, relative to analogue 28 from the HIR (KD = 1.18E-06) in the same position (Fig 2). KD values for analogues 1 and 13 were obtained in the peptide concentration range 0.28-7.5μM, whereas analogue 28 showed no binding until its concentration was increased to 22-169 μM. This indicated that analogues 1 and 13 were able to bind far more strongly to human insulin than analogue 28 from the HIR in the same binding position. Analogues 1 and 13 are highly conserved between *S*. *japonicum* and *S*. *mansoni* with 68-86% identity and have a 39-40% sequence homology with the HIR (Table 2).

**Table 2:** Details of the peptide analogues screened for human insulin binding activity.

Peptide	Sub-domain	Subunit divided	Insulin binding KD value	Concentration of peptides (μM)
1	L1 loop (same position as 1)	SjIR-1	2.24E-07	2.4-7.5
13		SjIR-2	5.51E-08	0.28-0.9
28		HIR	N/A	0.28-7.5
			1.18E-06	22-169
3	L1 loop (same position as 3)	SjIR-1	1.11E-08	0.22-0.75
15		SjIR-2	6.22E-07	13-40
29		HIR	N/A	0.22-40
			4.48E-05	33-224
21	L2 loop	SjIR-2	3.18E-05	65-260
20	FnIII-2	SjIR-2	6.76E-05	
10	FnIII-2 (same position as 9)	SjIR-1	5.44E-04	
22		SJIR-2	2.98E-05	
9 (positive control)		HIR	6.36E-05	

HIR, human insulin receptor.

Notably, analogue 3 (KD = 1.11E-08) from SjIR-1 and 15 (KD = 6.2E-07) from SjIR-2, which are located at the same sequence position as analogue 29 in the HIR (KD = 4.48E-05) (Fig 3), also bound strongly to insulin. KD values for analogues 3 and 15 were obtained in the peptide concentration range 0.22-40 μM, while there was no binding response detectable with analogue 29 until its concentration was increased to 33 μM, and measured over the concentration range of 33-224 μM. This indicated that analogues 3 and 15 were able to bind far more strongly to human insulin than analogue 29 from the HIR in the same binding position. Interestingly, analogue 3, with high sequence identity (84%) to SmIR-1, showed the strongest insulin binding capacity (1000-fold lower in KD value than that in HIR) and had the lowest sequence similarity level (27%) to the HIR (analogue 29), suggesting it may represent a highly specific insulin binding site in schistosomes.

The similar insulin binding positions in the HIR and the SjIRs, and the relatively high sequence identity (39-41%) for analogues 1, 13, 15 (Table 2) between the HIR and the SjIRs for analogues 28 and 29, argue against their further development as peptide drug or vaccine targets. In contrast, analogue 3 exhibited the strongest insulin binding capacity and has the lowest level of sequence identity (27%) with the HIR (analogue 29), suggesting it may represent a potential intervention target for interrupting or blocking the binding between host insulin and schistosome IRs. However, due to the presence of multiple insulin binding sites on the SjIRs, blocking only a single insulin binding site would likely be insufficient to disrupt the parasite’s insulin pathway.

Overall, the results of the binding assays showed that the SjIRs have a stronger competitive capacity to bind host insulin (more than 10 fold lower in KD value) than HIR by sharing the same major insulin binding sites I (analogues 1 and 13) and II (analogues 3 and 15) in SjIR-1 and 2, respectively. It has been well established that a competitive relationship amongst growth factors occurs ubiquitously in mammalian cells allowing them to interact with different growth factor receptors containing a similar structure [32]. However, weaker binding induced by non-specific interactions may not activate or phosphorylate downstream proteins. In this respect, we have demonstrated that the binding between human insulin and the SjIRs activated downstream signalling transduction in the insulin pathway, a process which has been shown to play an important role in regulating glucose uptake and fecundity in schistosomes [13]

In addition to insulin binding sites I and II from SjIR-1 and 2, we also identified analogue 10 from SjIR-1 and analogues 20, 21 and 22 from SjIR-2 which presented with binding activity but at a much weaker level when compared with analogues from binding sites I and II (Table 2). Analogue 10, from SjIR-1, located at the same position as the positive control (analogue 9, KD = 6.36E-05) but derived from the HIR (Table 1), exhibited moderate insulin binding activity (KD = 5.44E-04, Table 2). Furthermore, we determined that analogue 21 (KD=3.18E-05) derived from the L2 loop of SjIR-2 and analogues 20 (KD=6.76E-05) and 22 (KD = 3.0E-05), derived from the FnIII-2 sub-domains of SjIR-2, were *S*. *japonicum*-specific insulin binding peptides (Table 2). Sequence analysis showed that analogue 21 has a 28% identity with HIR but there is no similarity between analogue 20 (or 22) and HIR in the same sequence position.

However, HIR may compete with the SjIRs in binding human insulin in other positions. We thus designed analogues 2, 7 and 8 derived from SjIR-1 and analogues 14, 16 and 18 derived from SjIR-2 at the same locations as HIR sequences, which have been shown to be important for insulin binding to the HIR [15] [23,24] (Fig 1, Table 1). However, no specific binding occurred between human insulin and these parasite analogues in subsequent binding assays, further suggesting *S*. *japonicum* has its own specific insulin binding sites which are different to those essential for insulin binding in the HIR.

### Structural analysis

**Fig 4:**
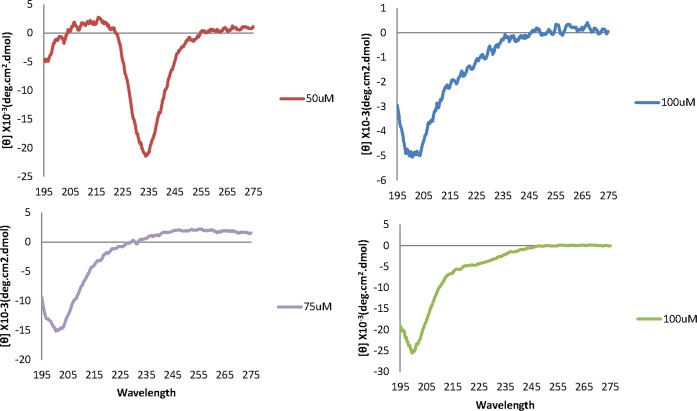
Circular dichroism spectra for peptide analogues 1 (top left), 3 (top right), 13 (bottom left), and 15 (bottom right).

**Fig 5:**
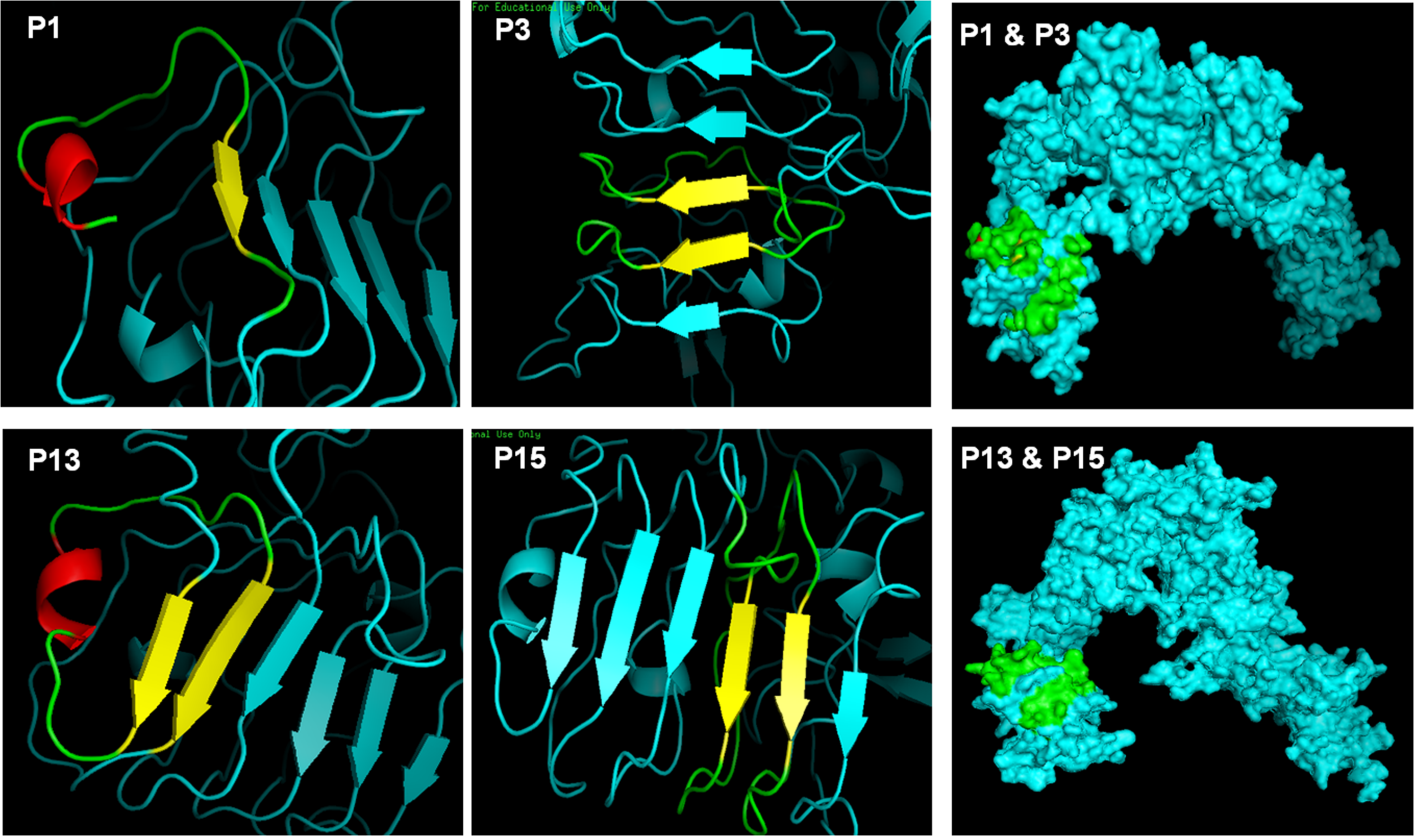
Molecular modelling of the SjIR-1 and SjIR-2 proteins showing the location of peptide analogues 1, 3, 13, and 15. The top panel shows the location on the SjIR-1 protein of analogues 1 (left), 3 (middle), and 1 and 3 combined (right). The bottom panel shows the location on the SjIR-2 protein of analogues 13 (left), 15 (middle), and 13 and 15 combined (right). Cyan represents the protein with peptide analogues identified as yellow (β-sheet), red (coiled-coil) or green (the secondary structure is absent).

To ensure the analogues we designed contained predicted secondary structures that can adopt a similar conformation to the native protein target, secondary structure analysis of analogues 1, 3, 13, and 15 was carried out using circular dichroism and modelling prediction tools [11]. Accordingly, analogue 1 adopted a mostly β-sheet secondary structure while analogues 3, 13 and 15 had a random coil secondary structure based on the circular dichroism analysis (Fig 4). Using modelling prediction tools, all four analogues (1, 3, 13, and 15) were located on the surface of the protein (Fig 5). The predicted conformation of analogues 3 and 15 within the SjIR-1 and 2 proteins, respectively, was primarily β-sheet, whereas the predicted conformation of analogues 1 and 13 within the SjIR-1 and 2 proteins, respectively, was primarily β-sheets with a small fragment forming a coiled-coil internally for analogue 13 and at the N-terminus for analogue 1 (Fig 5). Maintaining the predicted secondary structure of the SjIR peptide analogues could be essential for determining their interactions with insulin, but further studies specifically looking at the effect of SjIR peptide secondary structure effect would be necessary to confirm this [33]. For example, peptide epitopes, J8 and J14, identified from the Group A Streptococcus M protein, have been shown to only generate protective antibodies when the peptides are in the native alpha-helical formation (as occurs in the native M protein) [34].

### ELISA and Western blot analysis

Titres of mouse anti analogue 1, 3, 13, 15 antibodies were 1:256,000, 1:64,000, 1:8,000, 1:64,000, respectively. We found that none of the antisera bound consistently a *S*. *japonicum* native adult worm antigen preparation (SWAP) in ELISA or western-blot.

Although our structural modelling predictions indicated these four analogues are located on the surface of the SjIRs (Fig 5) and they have strong insulin binding affinity, the ELISA and Western blot analysis suggest that these peptides do not contain strong antigenic determinants which would argue against their further development as peptide-vaccine candidates. To further test the immunogenicity of analogues 1, 3, 13, and 15, we isolated spleen cells from mice infected with *S*. *japonicum* and then stimulated the cells with these peptides. T cell-mediated immune responses (including measuring levels of CD4/CD8 T cells, and IFNr, IL-4, IL-10 and IL-17) were determined using flow-cytometric staining, but no significant responses were detected, further reflecting the lack of antigenicity of these peptides (data not shown).

It is important to emphasize, however, that immunization with the ligand domain of the SjIR-1 (rSjLD-1) and SjIR-2 (rSjLD-2) fusion proteins, which include analogues 1, 3 and analogues 13, 15, respectively, induced a significant retardation in worm growth, depressed fecundity and the release of 75% immature intestinal eggs in a murine vaccine/*S*. *japonicum* cercarial challenge model [9,13]. Further, there is only 21-29% amino acid sequence identity between the SjLDs and HIR. Importantly, antibodies generated against the rSjLD-1 and rSjLD-2 fusion proteins were unable to recognize the HIR fusion protein [13]. The work described here reinforces the view that the antigenic determinants of the SjLD-1 and 2 proteins, coding for the ligand domain of the SjIRs, differ from the major insulin binding sites in HIR, adding further support for the development of recombinantly-derived SjIR protein vaccines.

## Conclusions

Our study has shown that analogues 1 and 3 derived from SjIR-1 and analogues 13 and 15 from SjIR-2 are responsible for the major insulin binding affinity in *S*. *japonicum*. Further, these peptides have more than 10 times stronger binding capacity for human insulin when compared with peptide analogues derived from the HIR in the same sequence positions. However, these analogues do not appear to contain major antigenic determinants as they generated poor antibody responses to native *S*. *japonicum* protein. Whereas this argues against the future development of these synthetic analogues as vaccine candidates, our study provides additional information about the IRs from these parasites, further supporting the development of a recombinantly-derived SjIR vaccine.

## Supporting Information

S1 Fig Alignment of amino acid sequences of extracellular regions of different insulin receptors using CLUSTAL W. The extracellular regions of SjIR-1 and 2 were aligned with those from insulin receptors in *S*. *mansoni* (SmIR-1 and 2), *E*.*multilocularis* (EmIR), *D*. *melanogaster* (DmIR) and *Homo sapiens* (HIR). Black boxes indicate identical amino acids and grey boxes denote sequence similarity. Peptides P13, P1, P28, P15, P3, P29, P21, P22, P10, P9 and P20 are shown boxed in different colours. These peptides all bound human insulin using the Octet RED system.
(PDF)

S2 Fig Quality control of peptide synthesis using analogue 13 as an example. The peptide (ADVRHSSSLTKLSRCTVIEGDLFIVFTR) was analyzed by RP-HPLC and MS. Left panel: RP-HPLC chromatogram of analogue **13;** Right panel: MS chromatogram of analogue **13**.
(TIF)

S1 Table Characterization of peptides 1-22 by RP-HPLC and ESI-MS and purification yields of those peptides. (PDF)
