# Supplementary figures and images for: Identification of Host Insulin Binding Sites on Schistosoma japonicum Insulin Receptors
Source: PLoS One. 2016 Jul 21;11(7):e0159704. doi: 10.1371/journal.pone.0159704 (PMC4956214; doi:10.1371/journal.pone.0159704)

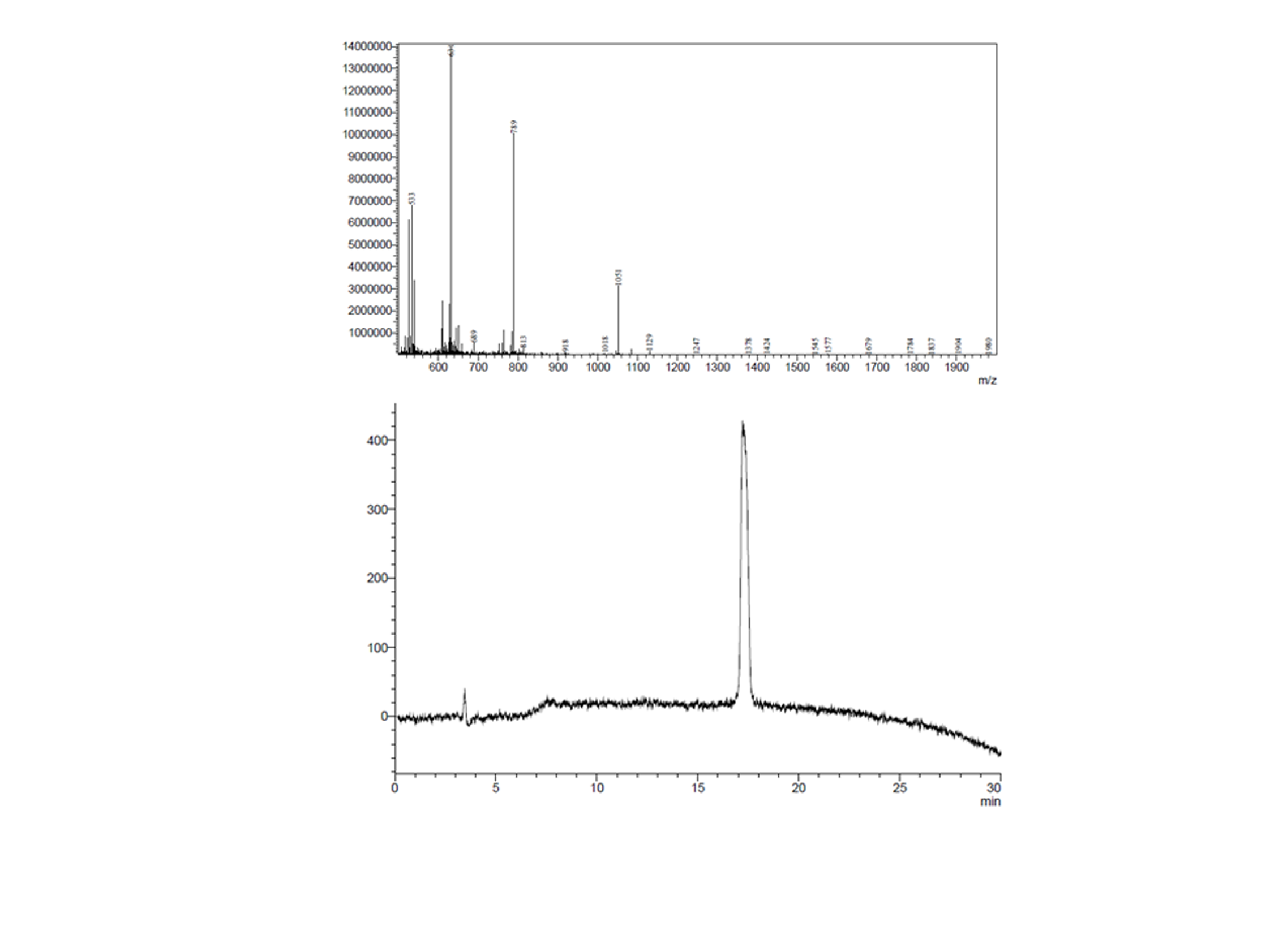

Supplement: S2 Fig — The peptide (ADVRHSSSLTKLSRCTVIEGDLFIVFTR) was analyzed by RP-HPLC and MS. Left panel: RP-HPLC chromatogram of analogue 13; Right panel: MS chromatogram of analogue 13. (TIF) [file pone.0159704.s002.tif]
