# Supplementary material for: Identification of Host Insulin Binding Sites on Schistosoma japonicum Insulin Receptors
Source: PLoS One. 2016 Jul 21;11(7):e0159704. doi: 10.1371/journal.pone.0159704 (PMC4956214; doi:10.1371/journal.pone.0159704)
Supplement: S1 Table — (PDF) [file pone.0159704.s003.pdf]

Table S1. Purification yields and characterisation of peptides **1-22** and **27-28** by RP-HPLC and MS.

Analogue	Yield mg (%)	RP-HPLC Rt (min)		MS		
		C18 Vydac column	C8 Vydac column	Ionisation	Calculated	Found
**1**	2.5 (18%)	14.5	14.6	$[M+H]^{+1}$, $[M+2H]^{+2}$, $[M+3H]^{+3}$	2756.1, 1379.5, 690.3	2758.4, 1379.5, 920.3
**2**	3.6 (32%)	14.5	-	$[M+2H]^{+2}$, $[M+3H]^{+3}$, $[M+4H]^{+4}$	1527.77, 1018.7, 764.3	1527.3, 1018.7, 764.3
**3**	3.9 (21%)	16.2	-	$[M+3H]^{+3}$, $[M+4H]^{+4}$, $[M+5H]^{+5}$, $[M+6H]^{+6}$, $[M+7H]^{+7}$	1410.7, 1062.3, 850.0, 708.5, 607.4	1416.3, 1062.4, 846.9, 706.7, 607.5
**4**	2.2 (37%)	11.2	9.6	$[M+H]^{+1}$, $[M+2H]^{+2}$	1588.89, 795.0	1589.9, 796.0
**5**	3.8 (40%)	13.0	14.8	$[M+H]^{+1}$, $[M+2H]^{+2}$, $[M+3H]^{+3}$	1535.81, 768.5, 512.7	1536.0, 769.0, 516.2
**6**	3.5 (35%)	15.2	15.5	$[M+H]^{+1}$, $[M+2H]^{+2}$, $[M+3H]^{+3}$	1691.2, 846.6, 564.7	1692.9, 846.8, 565.1
**7**	2.4 (19%)	17.5	17.8	$[M+1H]^{+1}$, $[M+2H]^{+2}$	1520.72, 761.0	1521.0, 761.2
**8**	4.8 (44%)	17.5	-	$[M+2H]^{+2}$, $[M+3H]^{+3}$	822.96, 548.3	822.9, 549.0
**9**	8.0 (51%)	13.2	13.5	$[M+2H]^{+2}$, $[M+3H]^{+3}$, $[M+4H]^{+4}$	959.5, 639.7, 480.0	959.6, 640.1, 480.3
**10**	4.2 (48%)	13.5	14.0	$[M+2H]^{+2}$, $[M+3H]^{+3}$	874.90, 583.3	875.8, 583.6
**11**	6.0 (40%)	13.6	-	$[M+2H]^{+2}$, $[M+3H]^{+3}$, $[M+4H]^{+4}$	966.08, 644.4, 483.6	965.9, 644.3, 483.5
**12**	4.8 (28%)	12.1	-	[M+H]+1, [M+2H]+2, [M+3H]+3, [M+4H]+4	1917.02, 959.5, 640.0, 480.3	1916.9, 959.1, 640.1, 480.3

Supplementary Table S1 (cont'd). Purification yields and characterisation of peptides **1-22** and **27-28** by RP-HPLC and MS.

Analogue	Yield mg (%)	RP-HPLC Rt (min)		MS		
		C18 Vydac column	C8 Vydac column	Ionisation	Calculated	Found
**13**	2.4 (18%)	17.5	-	$[M+2H]^{+2}$, $[M+3H]^{+3}$, $[M+4H]^{+4}$, $[M+5H]^{+5}$, $[M+6H]^{+6}$	1576.3, 1051.2, 788.7, 631.1, 526.1	1576.9, 1051.6, 781.9, 635.0, 521.6
**14**	4.9 (28%)	17.9	18.0	$[M+2H]^{+2}$, $[M+3H]^{+3}$	1538.0, 1025.7	1537.7, 1026.2
**15**	3.3 (19%)	17.5	-	$[M+2H]^{+2}$, $[M+3H]^{+3}$	2104.5, 1403.0	2150.4, 1404.1
**16**	3.5 (38%)	19.8	18.5	$[M+H]^{+1}$, $[M+2H]^{+2}$	1994.28, 998.0	1995.3, 998.6
**17**	4.0 (30%)	9.0	9.5	$[M+H]^{+1}$, $[M+2H]^{+2}$	1656.65, 829.0	1657.3, 829.2
**18**	4.2 (28%)	17.5	17.5	$[M+H]^{+1}$, $[M+2H]^{+2}$	1882.23, 942.0	1882.6, 942.1
**19**	5.0 (28%)	18.0	11.3	$[M+H]^{+1}$, $[M+2H]^{+2}$	1413.47, 708.2	1414.3, 708.1
**20**	6.6 (35%)	12.0	12.1	$[M+H]^{+1}$, $[M+2H]^{+2}$	1666.84, 834.4	1667.1, 834.2
**21**	2.3 (24%)	13.0	13.2	$[M+H]^{+1}$, $[M+2H]^{+2}$	1711.07, 856.5	1712.2, 856.5
**22**	4.2 (39%)	14.5	14.5	$[M+H]^{+1}$, $[M+2H]^{+2}$, $[M+3H]^{+3}$	2190.39, 1097.4, 731.9	2192.8, 1096.8, 731.7
**28**	5.8 (21%)	19.3	20.6	$[M+2H]^{+3}$, $[M+2H]^{+4}$,	1152.0, 64.3	1151.8, 864.1
**29**	8.5 (25%)	7.7*	7.6	$[M+2H]^{+2}$, $[M+3H]^{+3}$, $[M+4H]^{+4}$, $[M+5H]^{+5}$	2374.3, 1583.0, 1187.5, 950.2	2374.5, 1582.29, 1188.3, 950.6

*C4 column used
