# Supplementary material for: Identification of Host Insulin Binding Sites on Schistosoma japonicum Insulin Receptors
Source: PLoS One. 2016 Jul 21;11(7):e0159704. doi: 10.1371/journal.pone.0159704 (PMC4956214; doi:10.1371/journal.pone.0159704)
Supplement: S1 Fig — The extracellular regions of SjIR-1 and 2 were aligned with those from insulin receptors in S. mansoni (SmIR-1 and 2), E.multilocularis (EmIR), D. melanogaster (DmIR) and Homo sapiens (HIR). Black boxes indicate identical amino acids and grey boxes denote sequence similarity. Peptides P13, P1, P28, P15, P3, P29, P21, P22, P10, P9 and P20 are shown boxed in different colours. These peptides all bound human insulin using the Octet RED system. (PDF) [file pone.0159704.s001.pdf]

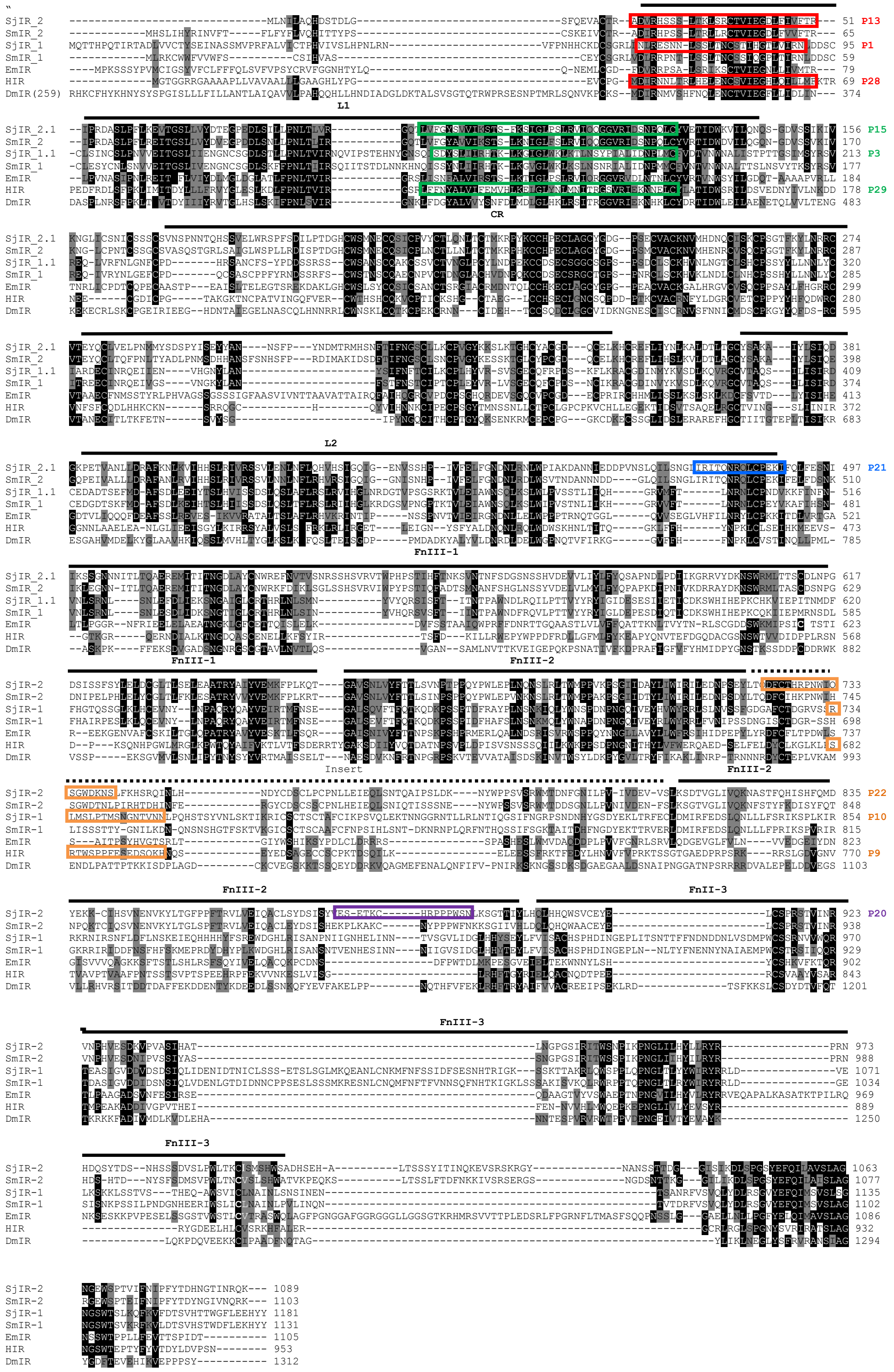
SjIR_2
SmIR_2
SjIR_1
SmIR_1
EmIR
HIR
DmIR
P13
P1
P28
L1
P15
P3
P29
CR
L2
P21
FnIII-1
FnIII-2
Insert
P22
P10
P9
FnII-3
P20
FnIII-3
